# Supplementary material for: Knowing the learning strategy is not enough to use it: Example in reading strategies for Japanese undergraduates
Source: PLoS One. 2023 Nov 21;18(11):e0293875. doi: 10.1371/journal.pone.0293875 (PMC10662718; doi:10.1371/journal.pone.0293875)
Supplement: S1 File — (ZIP) [file pone.0293875.s001.zip › SI1.pdf]

Knowing the learning strategy is not enough to use it: Example in reading strategies for Japanese undergraduates

Tsuyoshi Yamaguchi[1*]

1 Liberal Arts and Sciences, Nippon Institute of Technology, Minamisaitama-gun, Saitama Pref., Japan

* yamaguchi.tsuyoshi@nit.ac.jp

Supporting information

S1 Explanatory text regarding responses to participants. The presentation to participants will be made in Japanese and translated into English by Editage.

- I would now like to ask you about how you read explanatory texts such as papers and textbooks.
- For the reading methods mentioned in each sentence, please rate (1) how much you actually use it, (2) how effective you think the method is, and (3) how troublesome you think the method is.
- In addition, in terms of method (1), please answer whether you ‘A. have been aware of the existence of the method before’ or ‘B. did not realise it until you were asked in this questionnaire’ after actually using the method.
- Circle the number or letter to indicate how much each item applies to you.
- There are no right or wrong answers, so feel free to answer as you see fit.
