# Supplementary material for: Knowing the learning strategy is not enough to use it: Example in reading strategies for Japanese undergraduates
Source: PLoS One. 2023 Nov 21;18(11):e0293875. doi: 10.1371/journal.pone.0293875 (PMC10662718; doi:10.1371/journal.pone.0293875)
Supplement: S2 File — (ZIP) [file pone.0293875.s002.zip › SI2.pdf]

Knowing the learning strategy is not enough to use it: Example in reading strategies for Japanese undergraduates

Tsuyoshi Yamaguchi[1*]

1 Liberal Arts and Sciences, Nippon Institute of Technology, Minamisaitama-gun, Saitama Pref., Japan

* yamaguchi.tsuyoshi@nit.ac.jp

Supporting information

S2 Scaling of participants' responses to reading strategy item. The presentation to participants will be made in Japanese and translated into English by Editage.

- Completely disagree
- Disagree
- Somewhat disagree
- Somewhat agree
- Agree
- Strongly agree
