# Supplementary material for: Knowing the learning strategy is not enough to use it: Example in reading strategies for Japanese undergraduates
Source: PLoS One. 2023 Nov 21;18(11):e0293875. doi: 10.1371/journal.pone.0293875 (PMC10662718; doi:10.1371/journal.pone.0293875)
Supplement: S3 File — (ZIP) [file pone.0293875.s003.zip › SI3.pdf]

Knowing the learning strategy is not enough to use it: Example in reading strategies for Japanese undergraduates

Tsuyoshi Yamaguchi[1*]

1 Liberal Arts and Sciences, Nippon Institute of Technology, Minamisaitama-gun, Saitama Pref., Japan

* yamaguchi.tsuyoshi@nit.ac.jp

Supporting information

S3 Reading strategy items. The presentation to participants will be made in Japanese and translated into English by Editage.

1. I read texts while asking questions that test the extent to which I understand the content.
2. I read texts thoroughly until I understand their meaning.
3. I add notes such as comments and summaries of the content.
4. I read while making connections to things I am familiar with.
5. I try to memorise important words instead of the meaning.
6. I read while thinking about the structure of sentences.
7. I identify words or terms I do not understand.
8. I write down important parts.
9. I pay attention to connections between sentences.
10. I read while clarifying the meaning of words.
11. I underline important parts.
12. I read parts I do not understand slowly.
13. I read while coming up with specific examples.
14. I try to understand what the content is referring to.
15. I tentatively memorise important sentences before understanding them.
16. I adjust my reading speed depending on how difficult the text is.
17. I memorise difficult words or content verbatim without understanding them.
18. I ask myself whether I understand the text.
19. I read while anticipating what comes next.
20. I focus on parts I do not understand or find difficult.

21. I read while making comparisons to what I already know.
22. I write summaries for each paragraph.
23. If there is something I do not understand, I think about where in the text I started to not understand the content and reread from there.
24. I reread texts to memorise them.
25. I read while thinking about whether the content is accurate.
26. I read while making sure I know the meaning.
27. I predict the overall idea based on the context.
28. I try to make connections between what I am reading and my knowledge.
